# Evaluation of Acetylcholinesterase Biosensor Based on Carbon Nanotube Paste in the Determination of Chlorphenvinphos

**DOI:** 10.1155/2011/974216

**Published:** 2011-05-02

**Authors:** A. C. Oliveira, L. H. Mascaro

**Affiliations:** ^1^Instituto de Química, Universidade Federal de Uberlândia, 593, 38400-902 Uberlândia, MG, Brazil; ^2^Departamento de Química, Universidade Federal de São Carlos, 676, 13560-970 São Carlos, SP, Brazil

## Abstract

An amperometric biosensor for chlorphenvinphos (organophosphorus pesticide) based on carbon nanotube paste and acetylcholinesterase enzyme (CNTs-AChE biosensor) is described herein. This CNTs-AChE biosensor was characterized by scanning electron microscopy (SEM) and electrochemical impedance spectroscopy (EIS). The SEM result shows the presence of CNTs and small lumps, due to the enzyme AChE, which has a type of cauliflower formation. From EIS analysis is possible to observe increased *R*_tc_ for CNTs-AChE biosensor when compared to the carbon nanotube paste electrode for the reaction [Fe(CN)_6_]^4−/3−^. Using a chronoamperometric procedure, a linear analytical curve was observed in the 4.90 × 10^−7^–7.46 × 10^−6^ M range with limit of detection of 1.15 × 10^−7^ M. The determination of chlorphenvinphos in the insecticide sample proved to be in agreement with the standard spectrophotometric method, with a 95% confidence level and with a relative error lower than 3%. In this way, the CNTs-AChE biosensor presented easy preparation, fast response, sensitivity, durability, good repeatability, and reproducibility.

## 1. Introduction

 Carbon nanotubes (CNTs) consist of cylindrical graphene sheets with nanometer diameters and present many unique characteristics, such as large ratio of surface area to mass, high electrical conductivity, and remarkable mechanical strength. CNTs include both single-walled and multiwalled structures. Single-wall CNTs (SWCNTs) are comprised of cylindrical graphite sheets of nanoscale diameter capped by hemispherical ends. Multi-wall CNTs (MWCNTs) are comprised of several to tens of incommensurate concentric cylinders of these graphitic shells with a layer spacing of 0.3–0.4 nm. MWCNTs tend to have diameters in the 2–100 nm range and can be considered as a mesoscale graphite system [1].

Since their discovery in 1991 [2], extensive applications have been found in the physical, chemical, and material science fields. The advantages of CNTs, such as their high surface area, favorable electronic properties, and electrocatalytic effect, have recently attracted considerable attention for the construction of electrochemical biosensors. Electrochemical biosensors, particularly enzyme electrodes, have benefited greatly from the ability of CNT-based transducers to promote the electron-transfer reactions of enzymatically generated species, such as hydrogen peroxide [3, 4] or NADH [5], and from the resistance to surface fouling of transducers. 

Electrochemistry is a powerful tool for real-time detection compared to fluorescence and spectrophotometry, which involves expensive detection systems. A combination of enzymatic reactions with the electrochemical method of monitoring electroactive enzymatic products allowed the development of enzyme-based electrochemical biosensors for sensitive and rapid determination of important environmental pollutants. 

Chlorphenvinphos is an organophosphate (OP) compound used as an agricultural and household pesticide [6]. The toxic action of chlorphenvinphos is based on its ability to irreversibly modify the catalytic serine residue in acetylcholinesterase (AChE) and effectively prevent nerve transmission by blocking breakdown of the transmitter choline [7]. For these reasons, the rapid determination and reliable quantification of trace levels of chlorphenvinphos are important for health and environmental reasons. Biosensors based on the inhibition of AChE have been widely used for the detection of OP compounds. The methodology involves the measurement of the uninhibited activity of the enzyme, followed by an incubation period for the reaction between enzyme and the inhibitor, and the measurement of enzyme activity after the inhibition. 

Recently, CNTs have been used for the construction of biosensors based on the inhibition of AChE activity for the determination of OP compounds [8]. The biosensor was prepared by mixing CNTs with mineral oil. Such composite electrodes combine the ability of CNTs to promote electron-transfer reactions with the attractive advantages of paste electrode materials. These materials allow easy immobilization, reproducible electrochemical behavior, and useful physical characteristics [9–11]. This study describes the preparation and application of acetylcholinesterase biosensors based on carbon nanotube paste (CNTs-AChE biosensor) in the amperometric detection of chlorphenvinphos. Compared to other analytical techniques, such as gas and liquid chromatography, enzyme-based electrochemical biosensors represent good selectivity, sensitivity, rapid responses, and reduced sizes in the determination of pesticide.

## 2. Experimental

### 2.1. Reagents and Solutions

All reagents were of analytical grade and used as received. The solutions were prepared with reverse osmosis water Gehaka (OS20 LX FARMA). The multiwall carbon nanotubes (MWCNT, 20–40 nm diameter, 5–15 *μ*m) came from Schenzhen Nanotech Port Co. Ltd. (Schenzhen, China). Acetylcholinesterase (AChE) (0.3 U mg^−1^) came from bovine erythrocytes. Acetylthiocoline iodide was purchased from Aldrich, and a 1.2 × 10^−2^ M stock solution was prepared in phosphate buffer pH 7.4. Chlorphenvinphos was purchased from Aldrich, and a 1.0 × 10^−3^ M stock solution was prepared in methanol.

### 2.2. Apparatus

The electrochemical measurements were performed using a model PGSTAT20 Autolab (Eco Chemie, Utrecht, Netherlands) potentiostat/galvanostat coupled to a personal computer and controlled with GPES 5.8 software. The electrochemical impedance spectroscopy (EIS) data were obtained using a PGSTAT30 Autolab (Eco Chemie, Utrecht, Netherlands) potentiostat/galvanostat controlled with FRA software. The electrochemical cell was assembled using a conventional three-electrode system: an Ag/AgCl in KCl (3 mol L^−1^) reference electrode, a Pt counter electrode, and a CNTs-AChE biosensor working electrode (1.2 mm diameter). All experiments were carried out at room temperature.

An NIR Cary Model 5G spectrophotometer was used for comparative method coupled to a personal computer and controlled with Cary Win UV software with a quartz cell (optical path of 1.00 cm). 

Scanning electron microscopy was performed in an FEG-VP Zeiss Supra 35 microscope, operated at 5 kV, at different magnitudes.

### 2.3. Preparation of the CNTs-AChE Biosensor

In previous studies carried out by our group, the most successful carbon nanotube paste was found using 6/4 (w/w) CNTs/Nujol. Therefore, this carbon nanotube paste composition was used in the present investigation for the construction of the biosensor using acetylcholinesterase enzyme. 

 The carbon nanotube paste electrode modified with AChE was prepared by mixing carbon nanotubes and Nujol in an agate mortar with pestle. Subsequently, 0.050 g of this mixture was modified by adding 6.75 mg of AChE 250 UN and mixing until a uniformly wetted paste was obtained. After this, the paste was packed into a glass tube (*ϕ* = 1.2 mm), and a copper wire was embedded in the paste for electrical connection.

### 2.4. Procedures

Square wave voltammograms for 3.0 × 10^−4^ M acetylthiocoline iodide in phosphate buffer pH 7.4 and 0.14 U of acetylcholinesterase were obtained between 0 and 1.0 V at increments of 2 mV and a frequency 50 Hz in order to evaluate the oxidation process of thiocholine, the product of the enzymatic reaction using carbon nanotube paste electrode 60% (w/w). Amperometric analysis was performed using acetylthiocoline iodide 3.0 × 10^−3^ M in phosphate buffer pH 7.4 and CNTs-AChE biosensor. The potential applied was 0.3 V. 

 For the determinations of chlorphenvinphos samples, experiments of standard addition were carried out using the amperometric method. Insecticide samples were prepared by dissolving in methanol and diluting to volume with phosphate buffer pH 7.4. After this, an aliquot of this solution was transferred into the cell and amperometric measurements were recorded in triplicate. Next, three successive additions of 100 *μ*L of a standard 2.5 × 10^−4^ M chlorphenvinphos solution in phosphate buffer pH 7.4 were performed. After each addition, amperometric measurements were recorded and the mean current was determined.

A spectrophotometric method for the determinations of chlorphenvinphos was used to compare the obtained analytical results with the proposed method.

The electrochemical impedance spectroscopy (EIS) data were obtained for frequencies from 10,000 Hz to 0.01 Hz at an amplitude of 10 mV. The impedance spectra were obtained within the ac potential, in 5 mM potassium ferricyanide in 0.5 M KCl, in the format of Nyquist plots.

## 3. Results and Discussion

CNTs have been known to promote electron transfer reactions due to their electronic structure, high electrical conductivity and redox active sites [12, 13]. The electrocatalytic action of CNTs facilitates low-potential measurements of the product of enzymatic reaction. For this reason, the CNTs-AChE biosensor was prepared without introduction of redox mediators (RM), which are able to shuttle electrons between the active site of redox enzymes, and an electrode replacing the natural cosubstrate of the enzyme. The CNTs-AChE biosensor combines the ability of carbon nanotube paste to promote electron-transfer reactions with the attractive advantages of paste electrode materials. These materials allow easy enzyme immobilization, reproducible electrochemical behavior and useful physical characteristics.

The use of CNTs as a matrix for immobilization exhibits advantages for chemically modified electrodes, primarily in the diversity of preparation methods for sensors and biosensors. As CNT matrices are effective in the immobilization process as transducer material, they are used together in the composite production as carbon paste. The coupling of the biocatalytic material and the electrode surface can be promoted through the interaction between the functional groups of the materials and the enzyme through the terminal amino acids [14]. For this reason, the CNTs-AChE biosensor was prepared without introduction of solid support for immobilization of the AChE. 

**Figure 1:**
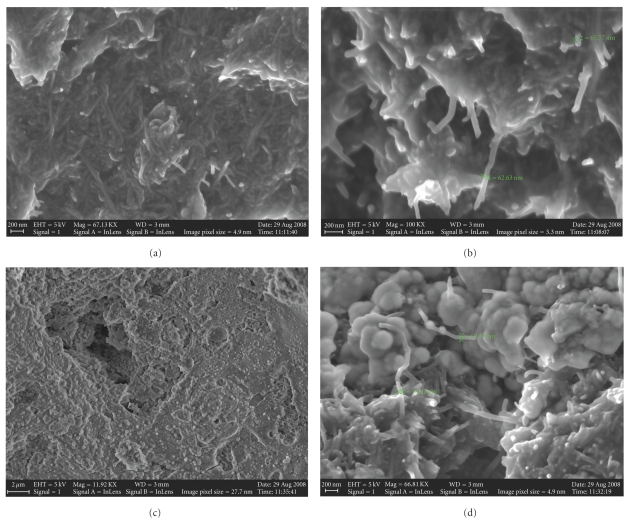
Scanning electron micrographs for carbon nanotube paste electrode (60% w/w) (a and b) and CNTs-AChE biosensor (c and d). Scales: (a) 200 nm, (b) 200 nm, (c) 2 *μ*m, and (d) 200 nm.

 Micrographies of the carbon nanotube paste electrode (60% w/w) and CNTs-AChE biosensor surfaces after polishing with 600 grit sandpaper are presented in Figure 1 for different magnitudes. The comparison of images shows a significant difference in the morphology of materials. In Figure 1(d), it is possible to observe the presence of CNTs and small lumps, due to the enzyme AChE, which has a type of cauliflower formation.

**Figure 2:**
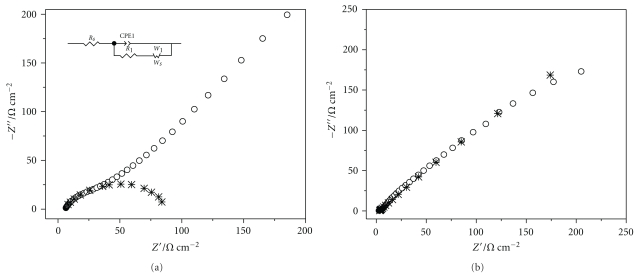
Typical Nyquist plots: (a) carbon nanotube paste electrode (60% w/w), (b) CNTs-AChE biosensor in 5.0 mM K_3_[Fe(CN)_6_] in 0.5 M KCl solution. Insert: the proposed Randle's model.

The EIS experiments, from Nyquist plots, allow the obtainment of charge transfer resistance values for the electrode process being studied. As such, the experiments were carried out in the following conditions: (A) carbon nanotube paste electrode (60% w/w), at 0.346 V (ac potential), (B) CNTs-AChE biosensor, at 0.192 V, all 5 mM Fe(CN)_6_^3−^ in 0.5 M KCl solution. All responses (Figure 2) presented typical semicircles at high frequencies and a straight line at low frequencies, corresponding to kinetic and diffusional processes, respectively.

To fit the EIS data, the corresponding spectra were modeled using Randle's equivalent circuits of mixed kinetic and diffusional control (insets in Figure 2(a)), where *R*_*s*_ is the electrolyte resistance, *C* the interface capacitance, and *R*_ct_ the charge-transfer resistance (domain of the kinetic control), resulting from the diffusion of Fe(CN)_6_^3−^ towards the electrode surface from the bulk of the electrolyte. As evidenced in the Nyquist plots, the simulated symbols (cross) based on the model agree with the experimental results. The estimated parameters obtained by assuming Randle's model are listed in Table 1. 

The value of *R*_tc_ for the CNTs-AChE biosensor increased threefold compared to the carbon nanotube paste electrode for the reaction [Fe(CN)_6_]^4−/3−^. This is probably due to the presence of the AChE enzyme at the electrode surface, as seen in micrographs, which is not conductive.

### 3.1. Optimization of the CNTs-AChE Biosensor Response

**Figure 3:**
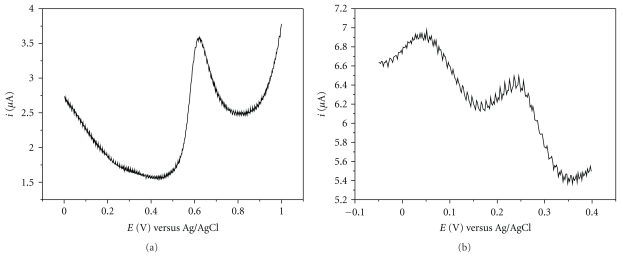
Square wave voltammograms obtained at the 60% (w/w) carbon nanotube paste electrode using 3.0 × 10^−4^ M acetylthiocholine iodide solution in phosphate buffer pH 7.4 (a) and thiocholine (b). Δ*E*_*p*_ = 50 mV, Δ*E*_*s*_ = 2 mV, and *f* = 50 Hz.

In the current study, the voltammetric characteristics of acetylthiocholine iodide (substrate) and thiocholine (enzymatically-generated product) on the carbon nanotube paste electrode were investigated by square wave voltammetry in phosphate buffer. Typical square wave voltammograms are shown in Figure 3. Two oxidation processes (peaks I and II) of thiocholine are observed at 0.045 and 0.250 V (versus Ag/AgCl) as observed by Liu et al. [13] for a glassy carbon electrode modified with carbon nanotube film. The anodic oxidation peak of acetylthiocholine iodide was observed at 0.620 V and its oxidation process begins at 0.500 V. As the potential of oxidation of thiocholine and the initial oxidation of acetylthiocholine iodide are close to one another, a study on the working potential was achieved using chronoamperometry. 

**Figure 4:**
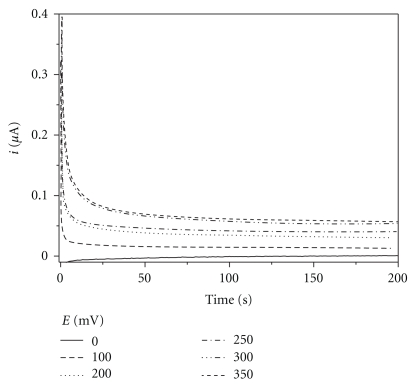
Effect of the applied potential on the oxidation of thiocholine in phosphate buffer pH 7.4 using 60% (w/w) carbon nanotube paste electrode.

The optimum potential for biosensor operation and current-time responses were obtained in thiocholine on a carbon nanotube paste electrode. The potential range evaluated was from 0 to 350 mV and the chronoamperometric responses obtained are presented in Figure 4. The maximum current responses were observed at 300 and 350 mV and the potential at 300 mV was selected for amperometric measurements of thiocholine with the CNTs-AChE biosensor. This potential was selected due to its greater substrate oxidation potential.

In order to optimize the CNTs-AChE biosensor's performance, the following experimental variables were investigated: substrate concentration and incubation time. The influence of substrate concentration on the biosensor response was studied, with the purpose of increasing the signal obtained for enzymatic reaction. Thus, the effect of the substrate concentration on the CNTs-AChE biosensor's response was investigated between 0.5 and 3.0 × 10^−3^ M acetylthiocholine iodide solution in phosphate buffer pH 7.4. The highest analytical signal was obtained at 3.0 × 10^−3^ M. 

Following this, the incubation time was studied ranging from 2 to 20 min. Incubation time is the time the biosensor remains immersed in the solution containing the pesticide and must be sufficiently extensive. The incubation time selected was 10 min. However, the maximum value of the inhibition was not 100%, which can likely be attributed to the binding equilibrium between pesticide and binding sites in the enzyme. Thus, these experimental conditions were selected for further experiments.  Table 2 summarizes the range over which each variable was investigated and the optimum value found in the optimization of the proposed method. 

The repeatability of the CNTs-AChE biosensor was determined from five different measurements in the same solution containing 3.0 × 10^−3^ M acetylthiocholine iodide in phosphate buffer pH 7.4. The electrode surface was renewed after each determination resulting in a mean peak current of 1.199 ± 0.013  10^−7^ A (*n* = 5). This result indicates good repeatability. Reproducibility was investigated considering three biosensors prepared independently. An acceptable reproducibility was obtained with a relative standard deviation of 10% for measurements carried out in 3.0 × 10^−3^ M of acetylthiocholine iodide in phosphate buffer pH 7.4. 

 The stability and life span of the biosensor are very important parameters in analytical determinations. For this reason, these parameters were investigated for the proposed biosensor in consecutive measurements without surfacing for over 20 days. When the CNTs-AChE biosensor was stored at −4°C with measurements taken every day for 20 days, no noticeable change was observed in the response obtained in 3.0 × 10^−3^ M of acetylthiocholine iodide in phosphate buffer pH 7.4.

### 3.2. Electroanalytical Method

The electrochemical determination of chlorphenvinphos was performed through the inhibition of the reaction of AChE with the substrate, acetylthiocholine, in order to allow the maximum inhibition to be achieved. The percentage of inhibition caused by chlorphenvinphos on the enzymatic activity of the biosensor was calculated using the following equation:

(1) $$\text{\%}I=\frac{\Delta I_{0}-\Delta I_{1}}{\Delta I}\times 100,$$

where Δ*I*_0_ and Δ*I*_1_ are the biosensor responses before and after the incubation procedure, respectively.

Under optimized conditions, a linear response between the percentages of inhibition as a function of chlorphenvinphos concentration was obtained in the range investigated: 4.90 × 10^−7^–4.76 × 10^−6^ M, with the limit of detection being 1.15 × 10^−7^ M. The curve was linear in the entire interval of chlorphenvinphos concentration, according to the following equation:

(2) $$\begin{array}{c} \%I=21.81\left(\%I\right)+4.91\;\left[\text{chlorphenvinphos}\right]\;\left(10^{-6}\;\text{M}\right),\\ r=0.995\;\left(n=6\right). \end{array}$$

**Figure 5:**
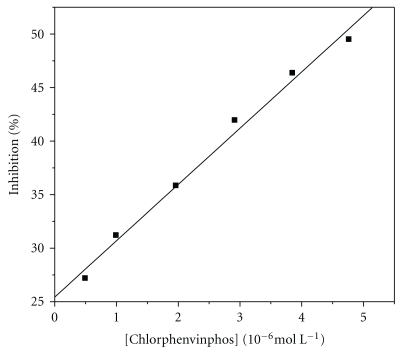
Analytical curve obtained with the CNTs-AChE biosensor for different concentrations of chlorphenvinphos in phosphate buffer pH 7.4 and 3.0 × 10^−3^ M AcSCh. Cell temperature is 30°C.

The result is presented in Figure 5, which shows the curve obtained with surface renewal between successive determinations. 

The favorable characteristics presented by the proposed biosensor allowed its application for the direct determination of chlorphenvinphos in real samples. Consequently, the performance of the CNTs-AChE biosensor was tested by applying it for the determination of chlorphenvinphos in the insecticide sample using the standard addition method. 

The results obtained by the CNTs-AChE biosensor were compared with those obtained by the spectrophotometric method. The results are summarized in Table 3. Applying a paired t-test to the results obtained by this procedure and spectrophotometric method, it was found that all results are in agreement at a 95% confidence level and with a relative error lower than 3%. These results therefore suggest that the CNTs-AChE biosensor is suitable for the determination of chlorphenvinphos in an insecticide sample.

## 4. Conclusions

In this paper, we have described the use of a CNTs-AChE biosensor for determination of chlorphenvinphos. The proposed system does not require any complicated immobilization procedure for the construction. The CNTs-AChE biosensor was prepared without the introduction of redox mediators. It is therefore concluded that the CNTs-AChE biosensor presents easy preparation, fast response, sensitivity, durability, good repeatability and reproducibility. Furthermore, this biosensor can be used in chronoamperometry for the determination of chlorphenvinphos in an insecticide sample, producing a relative error lower than 3%. The proposed method is, therefore, simple, fast and sensitive.

## Figures and Tables

**Table 1:** Summary of estimated EIS parameters obtained for the carbon nanotube paste electrode and CNTs-AChE biosensor.

Electrode	*n*	*R*_ct_ *t* (*Ω*)	*C*(*F*)
CNTPE	0.6472	90.5	4.5 × 10^−8^
CNTs-AChE biosensor	0.5330	3050.0	1.5 × 10^−6^

**Table 2:** Optimization of CNTs-AChE biosensor parameters.

Biosensor parameters	Range studied	Optimal value
Substrate concentration/10^−3^ mol L^−1^	0.5–3.0	3.0
Incubation time/min.	2–20	10

**Table 3:** Determination of chlorphenvinphos in insecticide sample using CNTs-AChE biosensor and spectrophotometric method.

Chlorphenvinphos/mg L^−1^		
CNTs-AChE biosensor	Spectrophotometric method	*E* ^*a*^/%
225	220	2.27

*E* ^*a*^: CNTs-AChE biosensor versus spectrophotometric method (CNTs-AChE biosensor—spectrophotometric method/spectrophotometric method) × 100%.
